# The chaperone GrpE mediates adhesion in *Mycoplasma bovis* and interactions with host extracellular matrix components and plasminogen

**DOI:** 10.1186/s13567-025-01619-4

**Published:** 2025-10-29

**Authors:** Xiangrui Jin, Huafang Hao, Ahmed Adel Baz, Shanyu Jin, Shimei Lan, Zhangcheng Li, Yifan Zhang, Yuefeng Chu, Shengli Chen

**Affiliations:** 1 State Key Laboratory for Animal Disease Control and Prevention, College of Veterinary Medicine, Lanzhou Veterinary Research Institute, Chinese Academy of Agricultural Sciences, Lanzhou University, Lanzhou, 730000 China; 2 Gansu Province Research Center for Basic Disciplines of Pathogen Biology, Lanzhou, 730046 China; 3 Key Laboratory of Veterinary Etiological Biology, Key Laboratory of Ruminant Disease Prevention and Control (West), Ministry of Agricultural and Rural Affairs, Lanzhou, 730046 China; 4 Botany and Microbiology Department, Faculty of Science, Al-Azhar University, Assiut, 71524 Egypt

**Keywords:** GrpE, *Mycoplasma*, extracellular matrix, plasminogen

## Abstract

*Mycoplasma bovis* (*M. bovis*) is a significant pathogen responsible for various clinical manifestations in cattle, including pneumonia, mastitis, and arthritis. The challenges associated with managing *M. bovis* infections include the absence of specific therapeutic agents, increasing antibiotic resistance and the absence of an effective commercial vaccine. The chaperone GrpE is a crucial component of mycoplasma bacteria. However, because there are few tools for genetic manipulation of *M. bovis*, the role of GrpE in pathogenicity is yet unknown. In this study, GrpE was universally present in the tested *M. bovis* strains, and it was localized on the surface membrane. We first demonstrated that GrpE plays a vital role in the ability of *M. bovis* to adhere to embryonic bovine lung (EBL) cells in a dose-dependent manner. Importantly, the presence of anti-GrpE serum specifically inhibited this adhesion. Additionally, GrpE was shown to bind various components of the host extracellular matrix (ECM), such as fibronectin, collagen IV, laminin, and vitronectin. This interaction not only facilitated adhesion but also promoted the activation of plasminogen into plasmin via tissue plasminogen activator (tPA). The significance of GrpE was further underscored in that disruption of the *grpE* gene markedly reduced the adhesion of *M. bovis* to EBL cells, whereas this ability was restored in the complemented strain *M. bovis* ∆*grpE*:*grpE*. In conclusion, GrpE represents a novel adhesion protein in *M. bovis* that is crucial for its interaction with the host ECM and plasminogen. This research highlights the importance of GrpE in the pathogenicity of *M. bovis* and suggests that targeting this protein could offer new avenues for vaccine development and therapeutic strategies against *M. bovis* infections.

**Supplementary Information:**

The online version contains supplementary material available at 10.1186/s13567-025-01619-4.

## Introduction

*Mycoplasma bovis* (*M. bovis*) is a globally prevalent pathogen that significantly affects the cattle industry and has serious economic repercussions. *M. bovis* poses a substantial threat to livestock health and is the primary cause of chronic pneumonia, arthritis, mastitis, otitis media, and meningitis in cattle [1, 2]. Its lack of a cell wall makes it resistant to antibiotics that target cell wall synthesis, rendering those treatment options ineffective. Consequently, there is an urgent need for effective antibiotics and a demand for viable disease control strategies [3, 4]. There are no effective commercial vaccines available for managing *M. bovis* infections, underscoring the need for intensified research efforts to combat this pathogen [5, 6].

As a small cell wall-deficient bacterium, *M. bovis* has unique structural characteristics that facilitate direct interactions with the membrane components of host cells. This property is pivotal for cytoadherence, which serves as a critical initial step in the colonization process and significantly contributes to the pathogenicity of mycoplasmas. This selective adhesion is vital for bacterial survival and colonization within the host environment. To achieve effective adherence and colonization, *M. bovis* expresses a range of adhesins, which latch onto extracellular matrix (ECM) components and plasminogen. Some of the adhesins identified in *M. bovis* include methylenetetrahydrofolate tRNA-(uracil-5-)-methyltransferase (TrmFO) [7], fructose-1,6-bisphosphate aldolase (FBA) [8, 9], α-enolase [10], mbfN [11], *Mycoplasma* immunogenic lipase A (MilA) [12], Mbov0503 [13] and lipoprotein LppA [14]. These adhesins, especially membrane proteins, may interact with various ECM components and plasminogen (Plg), thereby mediating cytoadherence. This interaction not only promotes the adhesion and invasion of pathogenic bacteria into host cells but is also involved in activating the immune response [14].

Molecular chaperones, commonly referred to as heat shock proteins (HSPs), have been identified in various bacterial species [15, 16]. GrpE is recognized as the sole nucleotide exchange factor for Hsp70 and organelles derived from bacteria and plays a crucial role in bacterial pathogenicity [17, 18]. Structurally, GrpE is characterized by an α-helix dimerization domain and a β-domain that facilitates its interaction with DnaK [17]. GrpE is involved in the adhesion and pathogenicity of group A *Streptococcus* (GAS) and *Streptococcus suis* [19, 20]. Moreover, there is a correlation between GrpE expression and stress conditions during the biofilm formation of certain bacteria [21–23]. Upon entering the host, bacteria are immediately exposed to different environments, including fluctuations in temperature, osmotic pressure, and pH. To effectively adhere, invade, or counteract these stressors, bacteria upregulate the expression of virulence factors and stress response proteins, which include HSPs. These are actively employed by bacteria to mount a robust compensatory response to adverse conditions, thereby ensuring their survival and pathogenicity [19, 24].

Preventive vaccines necessitate the expression of target antigens during the initial stages of infection, enabling recognition by the host immune system [25, 26]. GrpE is expressed by the immune system early in the infection process and provides protective immunity and substantial protection against *Mycobacterium tuberculosis* [26]. Furthermore, it presents a promising antigen target for the formulation of multiantigenic vaccines aimed at combating *Ureaplasma urealyticum* [27]. Importantly, the lack of efficient genetic tools for *Mycoplasma* results in a limited understanding of the function of GrpE in pathogenicity and the immune response. Therefore, the aim of this study was to investigate the potential mechanisms through which GrpE influences the adhesion of *M. bovis*, thereby enhancing our understanding of its pathogenicity. Understanding these mechanisms is crucial, as it provides important insights into how *M. bovis* establishes infection, which has significant implications for animal health. Furthermore, the findings from this research may position GrpE as a promising candidate for vaccine development, offering a strategic approach to prevent *M. bovis* infections.

## Materials and methods

### Strains and cells

*M. bovis* reference strain PG45, along with 6 clinical *M. bovis* isolates (Additional file 1), were cultivated in modified pleuropneumonia-like organisms (PPLO) medium at 37 °C, following previously established protocols [14]. *M. bovis* ∆*grpE*, identified from the transpon mutant library as previously described [28], was grown in modified PPLO medium supplemented with kanamycin (100 μg/mL). To establish the complementation strain *M. bovis* Δ*grpE:grpE*, the recombinant plasmid pIRR45-*grpE* was introduced into the ∆*grpE* mutant using the polyethylene glycol (PEG) method as previously described [28] and maintained in medium supplemented with 5 μg/mL tetracycline. DNA cloning and protein expression were conducted using *Escherichia coli* (*E. coli*) strains DH5α and BL21(DE3) (TransGen Biotech, China), respectively. Embryonic bovine lung (EBL) cells were cultured in Dulbecco’s modified Eagle’s medium (DMEM; Gibco, USA) supplemented with 10% fetal bovine serum (FBS; Gibco) and incubated at 37 °C with 5% CO_2_. The following day, the EBL cells were observed under a microscope (Olympus CKX31, Japan) for further experiments.

### DNA constructs

The pIRR45-*grpE* plasmid was constructed using an In-Fusion kit (Takara Bio, Japan) following the manufacturer's guidelines as previously outlined [28]. The *grpE* gene was amplified with the primers pIRR45-*grpE*-F/R (5′-tggactagtgcggccATGAGTTCGATGAATAAAGAAGAAG-3′; 5′-gacctgcaggcggccTTACTTATTGCTTTTTGCAATTGATTC-3′) and subsequently ligated to the complementary plasmid pIRR45 (pIRR45-F: 5′-GGCCGCCTGCAGGTCGACCATAT-3′; pIRR45-R: 5′-GCACTAGTCCAGATTTATATAACAAC-3′) via an In-Fusion kit. This construct was subsequently transformed into *E. coli* DH5α and subsequently verified by PCR.

### Bioinformatics analysis

Amino acid sequences that were homologous or similar to those of the GrpE protein in *Mycoplasma* were screened in the NCBI database by using the BLASTP program with default parameters. Sequence alignment was subsequently performed by the Clustal W method, and a phylogenetic tree was constructed using the neighbour-joining (NJ) method with 1000 bootstraps in MEGA 11 software.

### Recombinant protein expression and polyclonal antibody preparation

To express the recombinant proteins, the pET-30a-*grpE* plasmid was introduced into *E. coli* BL21 (DE3) cells. Following transformation, protein expression was induced using isopropyl β-d-thiogalactoside (IPTG, 0.05 mM), which was conducted in a shaking incubator at 16 °C for 16 h. The resulting protein was subsequently purified utilizing nickel-charged resin Ni–NTA (GenScript Biotech Corporation, China). The concentration of the purified protein was quantified using a bicinchoninic acid (BCA) assay kit (Beyotime, China). For polyclonal antibody production, the purified protein rGrpE was combined at a 1:1 (v/v) ratio with the adjuvant QuickAntibody-Mouse 5W (Biodragon, China) and administered intramuscularly to 6-week-old BALB/c mice at an interval of 21 days between two immunizations. Antisera were collected on day 35, the antibody titre was assessed via enzyme-linked immunosorbent assay (ELISA), and the specificity of the generated polyclonal antibody was evaluated through western blot analysis.

### Localization of GrpE in *M. bovis*

To determine the localization of GrpE, cytoplasmic and cell membrane proteins from *M. bovis* PG45 were extracted using a membrane extraction protein kit (Thermo Fisher, USA). The proteins, including whole bacterial protein, cell membrane protein, and cytoplasmic protein, were subsequently separated via 10% SDS-PAGE and transferred onto a polyvinylidene fluoride (PVDF) membrane (Amersham, USA). Afterwards, the membrane was blocked with 10% healthy goat serum (ZSGB-BIO, China) at room temperature for 2 h, after which it was incubated with anti-GrpE serum as the primary antibody (1:10 000) for 2 h at room temperature. The membrane was then washed 4 times with Tris-buffered saline Tween (TBST) and incubated for 1 h at room temperature with goat anti-mouse HRP-IgG (1:1000; Beyotime, China) as a secondary antibody. Imaging was conducted using a ChemiDoc imaging system (Bio-Rad, USA).

### Binding of EBL cell membrane protein detected by ELISA

EBL cell membrane protein was extracted utilizing a commercial cell membrane extraction kit (Thermo Fisher Scientific) following the manufacturer's instructions. ELISA was performed as previously described [28], with some modifications; a 96-well plate was coated with 800 ng of EBL cell membrane protein and subsequently blocked with 5% skim milk powder at 37 °C for 2 h. Various concentrations of rGrpE (800 ng to 3.125 ng) or rGrpE (800 ng) that had been preincubated with different dilutions of anti-GrpE serum or negative mouse serum from 1:10 to 1:2560 were then introduced and incubated at 37 °C for 1 h. Mouse anti-GrpE serum served as the primary antibody (1:1000 dilution), while goat anti-mouse HRP-IgG functioned as the secondary antibody (1:1000 dilution). Following 1 h of incubation at 37 °C, the OD was measured at a wavelength of 450 nm using an iMark microplate reader (Bio-Rad).

### Dot blot analysis

Twofold-diluted rGrpE protein and 6 × His polypeptides were applied to a nitrocellulose membrane (Solarbio, China) and allowed to dry at room temperature for 1 h. The membranes were subsequently subjected to blocking buffer (Beyotime, China) at room temperature for 1 h. Subsequently, the membranes were incubated overnight at 4 °C on a shaking platform with 10 μg of fibronectin (Sigma‒Aldrich), collagen IV (Sigma‒Aldrich), laminin (Roche), vitronectin (Sigma‒Aldrich), tissue-type plasminogen activator (tPA), or plasminogen (Cell Sciences). After incubation, the membranes were washed 5 times with TBST for 10 min each. Next, antibodies against fibronectin, collagen IV, laminin, vitronectin, tPA, or plasminogen (1:500; Abcam) were added, and the membranes were incubated at room temperature for 1 h. After another wash with TBST, either goat anti-mouse IgG-HRP (1:1000; Abcam) or goat anti-rabbit IgG-HRP (1:5000; Abcam) was added, and the membranes were incubated at room temperature for 1 h. Signal detection was carried out using ECL (Thermo Fisher Scientific) on an imaging detection system (Bio-Rad).

### ELISA for determination of ECM components

Fibronectin, collagen IV, laminin, and vitronectin (100 ng each) were added to 96-well plates. Subsequently, rGrpE protein was applied to the ELISA plates, which had been precoated with the ECM proteins, at various dilutions ranging from 800 ng to 3.125 ng, as previously outlined [14]. OD readings were recorded at a wavelength of 450 nm using an iMark microplate reader (Bio-Rad).

### Plasminogen activation assay

The rGrpE protein (20 μg/mL) was incubated with plasminogen (20 μg/mL) at 37 °C for 1 h. Subsequently, the mixture was transferred to a 96-well plate containing tPA (50 ng/mL, Sigma) or the lysine analogue aminoacetic acid ε-ACA (80 mM, Sigma), followed by an additional incubation at 37 °C for 15 min. After an incubation period, the D-Val-Leu-Lys para-nitroaniline hydrochloride substrate (0.5 mM, Sigma) was added, and the OD was monitored at 405 nm at 15 min intervals using an iMark microplate reader (Bio-Rad).

### Adherence and inhibition assays of rGrpE

rGrpE or 6 × His peptides (100 µg) were added to Opti-MEM (Gibco) and incubated overnight with EBL cells in a 24-well plate at 37 °C for 1 h. The EBL cells were subsequently fixed using 4% paraformaldehyde (PFA; Solarbio, China) at room temperature for 10 min, permeabilized with 0.1% Triton X-100 (Solarbio, China) for an additional 10 min, and blocked with 5% bovine serum albumin (BSA; Sigma) at 37 °C for 2 h. In the adhesion inhibition experiment, 100 µg of rGrpE protein was combined with mouse anti-GrpE serum or negative mouse serum (10 µL) in Opti-MEM and incubated at 37 °C for 1 h before the cells were fixed. Following a wash with phosphate-buffered saline (PBS), the cells were incubated with anti-GrpE serum or negative mouse serum (1:100) at 37 °C for 3 h and subsequently treated with goat anti-mouse Dylight 488 (1:1000; Thermo Fisher) for 45 min at 37 °C. EBL cell membranes and nuclei were stained with DiI (Beyotime, China) and 4′,6-diamidino-2-phenylindole (DAPI, Sigma), respectively. The cells were then observed with a Zeiss LSM 980 inverted laser scanning confocal microscope.

### *Mycoplasma* adhesion assay

EBL cells were cultured in 12-well plates and incubated overnight at 37 °C in a CO_2_ incubator. The cells were subsequently infected with *M. bovis* at a multiplicity of infection (MOI) of 1000 for 1.5 h at 37 °C. Following the infection period, the cells were washed 3 times with PBS and digested using TrypLE™ Express (Gibco), and the resulting dilutions were plated onto PPLO agar plates and then incubated at 37 °C with 5% CO_2_. The number of *M. bovis* colonies was counted after 3 days of incubation, and the adhesion percentage was calculated. In the adhesion inhibition experiment, anti-GrpE serum was preincubated with *M. bovis* at ratios of 1:10 and 1:20 at 37 °C for 1 h, while preimmune serum was used as a negative control.

### Statistical analysis

All the statistical analyses were conducted using unpaired *t* tests and one-way/two-way analysis of variance with GraphPad Prism 9.0 software. All values are expressed as the means ± standard deviations. Three independent experiments were conducted. Significant differences are indicated as follows: *p < 0.05, **p < 0.01, ***p < 0.001, and ns, not significant at *p* ≥ 0.05.

## Results

### Bioinformatics analysis

**Figure 1:**
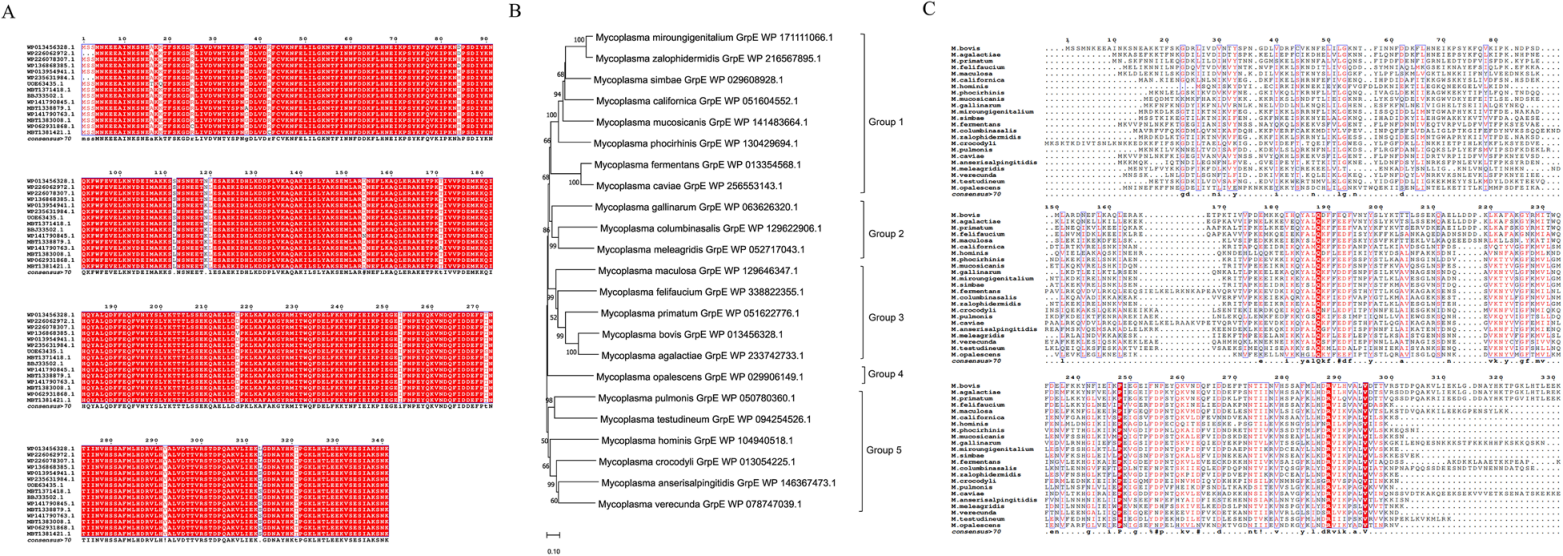
**Bioinformatics analysis of GrpE in** ***Mycoplasma*** **species. A** Genetic characteristics of *grpE* in *Mycoplasma bovis*. **B** Genetic characteristics of *grpE* in *Mycoplasma* species. A phylogenetic tree based on GrpE protein sequences was constructed using the neighbour-joining (NJ) method with 1000 bootstraps in MEGA 11 software. The phylogenetic tree was divided into five groups. **C** Multiple conserved sites of GrpE in Mycoplasma based on multiple alignment. Highly conserved residues are shown in red.

Genomic analysis revealed that the *grpE* gene is ubiquitously present across *M. bovis* isolates and represents one of the core genes of this species. The GrpE protein sequences among various *M. bovis* strains exhibited highly conserved amino acid sequences, with only a small number of mutations observed throughout the complete open reading frame, such as S112L, N119K, and L313S, which are present in very few isolates (Figure 1A). Moreover, according to phylogenetic analysis, GrpE can be categorized into five groups on the basis of its amino acid sequence in *Mycoplasma* species. *M. bovis* and *Mycoplasma agalactiae* are closely clustered together, and *Mycoplasma primatum*, *Mycoplasma felifaucium* and *Mycoplasma maculosa* form a large branch (Figure 1B). Multiple sequence alignment analysis revealed multiple conserved amino acid residues in GrpE. Specifically, the 174–298 aa region of the GrpE protein, along with residues Q188, P248, R288, and V295, was significantly conserved among *Mycoplasma species* (Figure 1C). These conserved sites may have comparable functional roles in diverse *Mycoplasma* species.

### Expression and purification of rGrpE

The recombinant expression plasmid pET30a-*grpE* was successfully constructed. Following induction with 0.05 mM IPTG for 16 h, the rGrpE protein was subsequently purified using Ni–NTA and predominantly expressed as a soluble protein of approximately 46 kDa in recombinant *E. coli* BL21(DE3), as confirmed by SDS-PAGE analysis (Additional file 2). Moreover, western blot analysis indicated that purified rGrpE was effectively recognized by mouse anti-6 × His monoclonal antibodies and anti-GrpE serum (Additional file 1).

### Surface localization of *M. bovis* GrpE and its adhesion to EBL cells

**Figure 2:**
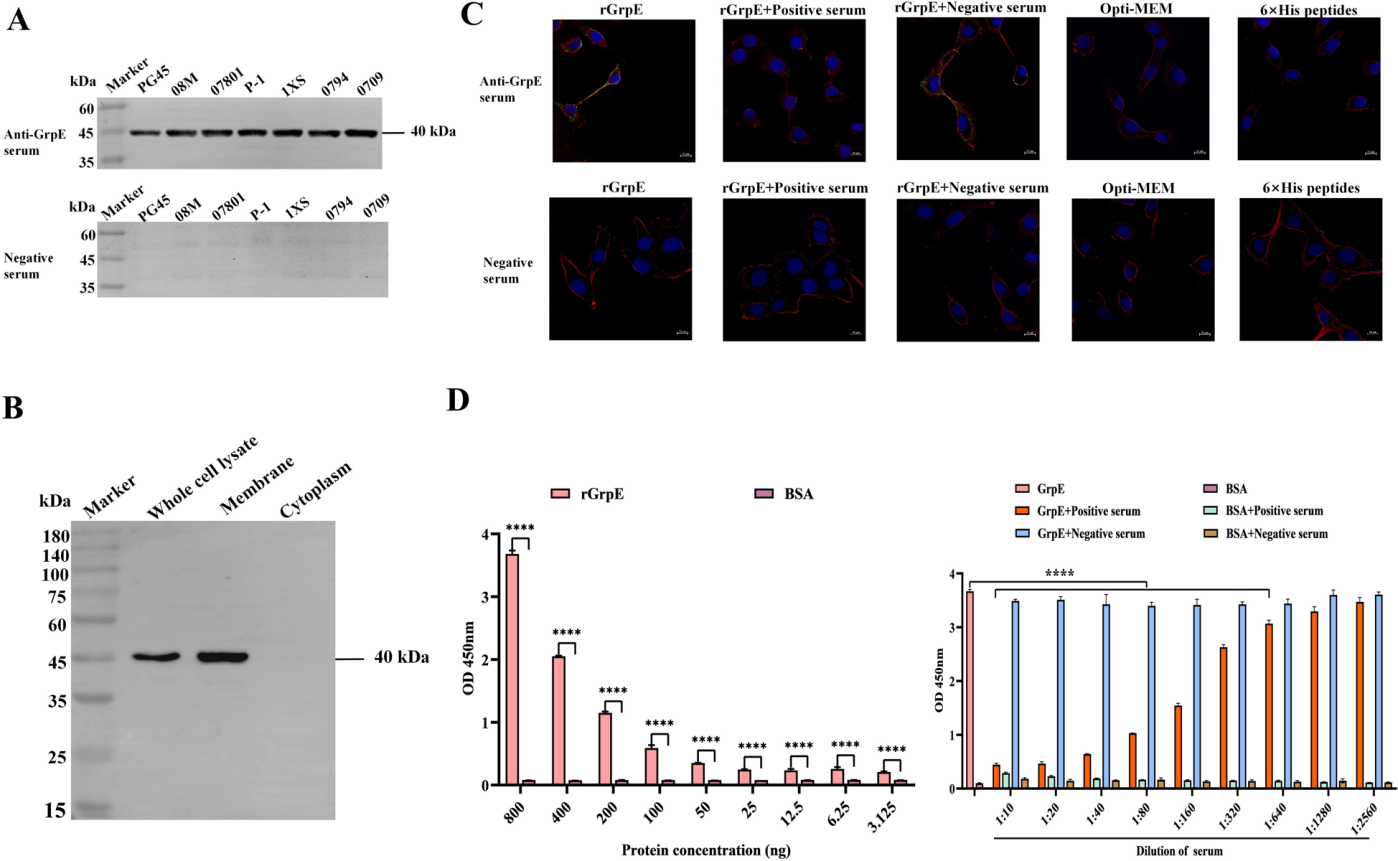
**Surface localization of GrpE in** ***M*** **. bovis and its adhesion to EBL cells. A** Evaluation of GrpE immunogenicity across different *M. bovis* strains. Whole-cell proteins from seven *M. bovis* strains were transferred to PVDF membranes, incubated with anti-GrpE serum or negative mouse serum (1:10 000), and subsequently detected using goat anti-mouse IgG-HRP (1:1000). **B** Membrane and cytoplasmic proteins of *M. bovis* PG45 were extracted, incubated with anti-GrpE serum (1:10 000), and then probed with goat anti-mouse HRP-IgG. **C** The adhesion of GrpE to EBL cells was observed by laser confocal microscopy. One hundred micrograms of rGrpE (6 × His peptides) was preincubated with positive or negative serum for 1 h and then tested with anti-GrpE serum or negative mouse serum (10 µL) and goat anti-mouse Dylight 488 (green, 1:1000). The cell membrane (red) and nucleus (blue) were labelled with DiI and DAPI, respectively (scale bar = 10 µm). **D** The binding of *M. bovis* GrpE and EBL cell membranes detected by ELISA. The EBL membrane protein (800 ng) was coated overnight and subsequently incubated with varying concentrations of rGrpE (ranging from 800 ng to 3.125 ng) for 1 h, after which anti-GrpE serum (1:1000) and goat anti-mouse IgG-HRP (1:1000) were added for TMB detection. For the inhibition test, EBL cell membrane protein (800 ng) was coated overnight, and rGrpE (800 ng) was incubated with anti-GrpE serum at different dilutions for 1 h. Subsequently, anti-GrpE serum (1:1000) and goat anti-mouse IgG-HRP (1:1000) were added for TMB detection. The values are presented as the mean ± standard error of three replicates (*p < 0.05, **p < 0.01, ***p < 0.001).

Western blot analysis confirmed the expression of GrpE among *M. bovis* PG45 and other clinical isolates (Figure 2A, Additional file 2). The localization of GrpE in the *M. bovis* PG45 strain was assessed through analysis of membrane proteins, cytoplasmic proteins, and whole-cell proteins in anti-GrpE serum. The results indicated that GrpE was predominantly localized in the membrane fraction of *M. bovis* (Figure 2B). This distribution highlighted the potential role of GrpE as a membrane-associated protein in the pathogenicity of *M. bovis*. Confocal microscopy revealed that rGrpE had a strong affinity for EBL cells, with a marked decrease in adhesion noted when rGrpE was pretreated with anti-GrpE serum, indicating that this serum significantly disrupted the binding of rGrpE, while the application of negative mouse serum did not significantly inhibit the adhesion process (Figure 2C). The 6 × His peptides did not adhere to EBL cells (Figure 2C). These findings underscored the specific interaction between GrpE and EBL cells. The binding interaction between GrpE and membrane extracts derived from EBL cells was examined using ELISA. Compared with the control group, the rGrpE-treated group exhibited dose-dependent binding affinity to the EBL cell membrane (Figure 2D). Furthermore, the binding of rGrpE to the EBL cell membrane was effectively inhibited by anti-GrpE serum at a dilution of 1:10 to 1:640, while the inhibitory effect of negative serum was not significant, suggesting that the interaction was specific and can be modulated by antibodies targeting GrpE.

### GrpE binds with host ECM components

**Figure 3:**
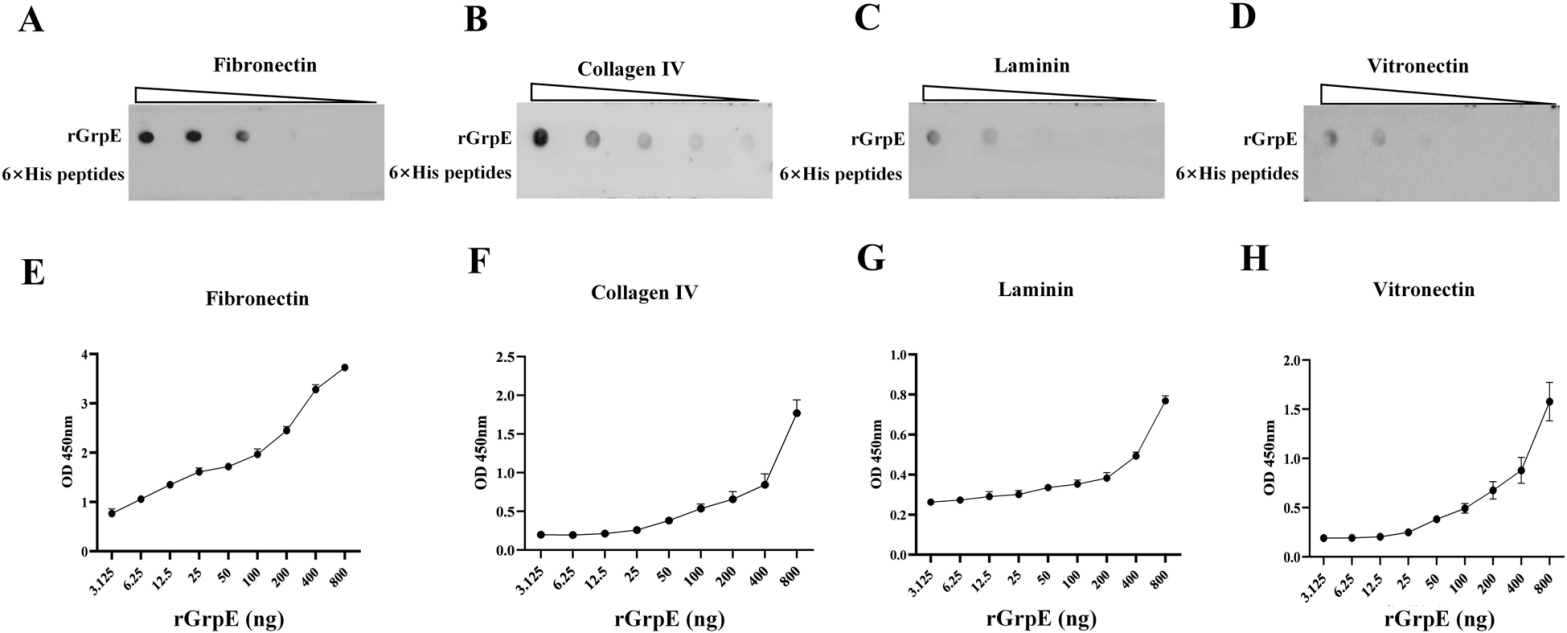
**Interaction of rGrpE with host extracellular matrix components**. **A**–**D** The detection of rGrpE binding to various host ECM components, including fibronectin, collagen IV, laminin, and vitronectin, using a dot blot assay, with a 6 × His peptides serving as the negative control. **E**–**H** The binding of rGrpE to fibronectin, collagen IV, laminin and vitronectin in a dose-dependent manner, as evidenced by ELISA.

To further investigate the adhesion properties of GrpE to host cells, we examined its potential interactions with ECM components. We employed both dot blot analysis and ELISA to evaluate the binding affinity of rGrpE for fibronectin, collagen IV, laminin, and vitronectin, with 6 × His peptides serving as a negative control. The findings confirmed that GrpE bound to fibronectin, collagen IV, laminin, and vitronectin in a dose-dependent manner (Figures 3A–H). This clear pattern of binding highlights the potential role of GrpE in mediating interactions with host cells.

### GrpE promotes the conversion of plasminogen to plasmin

**Figure 4:**
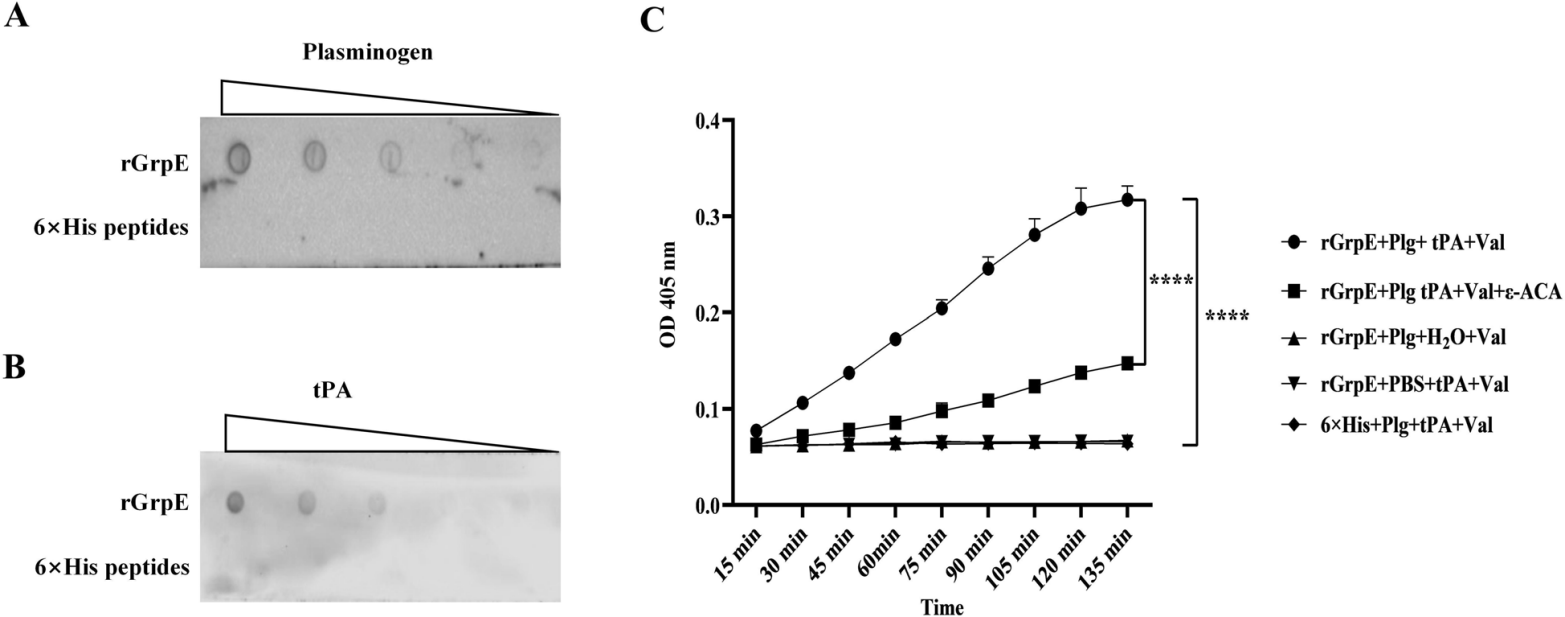
**Plasminogen activation activity mediated by GrpE**. **A**, **B** The binding affinity of rGrpE for both plasminogen and tPA, as assessed by dot blot analysis, with 6 × His peptides serving as a negative control. **C** GrpE facilitated plasminogen to plasmin in the presence of tPA. rGrpE was incubated with plasminogen for 1 h, tPA and the specific substrate d-Val-Leu-Lys p-nitroaniline dihydrochloride were added, and plasmin activity was measured as the optical density at 405 nm at 15 min intervals. ε-ACA was included as a lysine analogue.

GrpE exhibited a robust ability to bind plasminogen and tPA in a dose-dependent manner, as demonstrated by dot blot analysis (Figures 4A, B). The binding of GrpE to plasminogen by tPA was assessed using plasminase-specific chromogenic substrates, which allowed us to quantitatively measure plasminogen activation. The results indicated that wells containing rGrpE, plasminogen, and tPA displayed significantly higher OD readings at 405 nm than control wells containing 6 × His peptides did (Figure 4C). This increase underscores the effective conversion of plasminogen to plasminase facilitated by GrpE in the presence of tPA. Moreover, the addition of ε-ACA inhibited the capacity of GrpE to promote plasminogen activation, further supporting the specificity of the interaction between GrpE and plasminogen activation.

### Disruption of the grpE gene significantly reduces *M. bovis* adhesion to host cells

**Figure 5:**
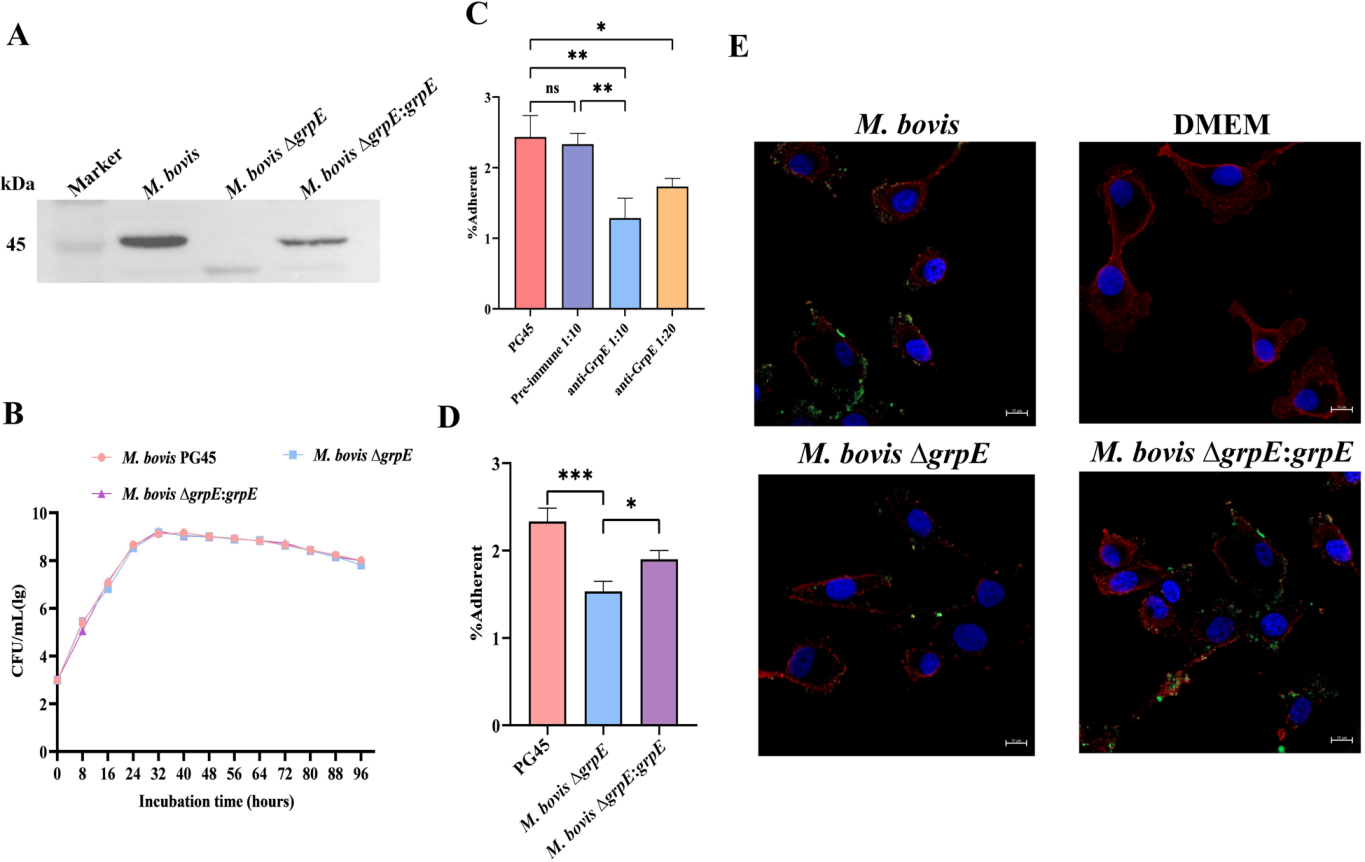
**Adhesion ability of GrpE**. **A** The expression of the GrpE protein in the *M. bovis* wild-type strain, *M. bovis* ∆*grpE* strain, and *M. bovis* ∆*grpE*:*grpE* strain detected by anti-GrpE serum. **B** Growth curves of the *M. bovis* wild-type strain PG45, *M. bovis* Δ*grpE*, and *M. bovis* Δ*grpE*:*grpE*. **C** The ability of *M. bovis* wild-type, *M. bovis* ∆*grpE*, and *M. bovis* ∆*grpE*:*grpE* strains to adhere to EBL cells was determined 1.5 h after infection, after which the cells were washed three times with PBS, digested with TrypLE™ Express, diluted and placed on mycoplasma agar plates for counting. **D** Inhibition of adhesion to *M. bovis* by GrpE. *M. bovis* was incubated with different concentrations of anti-GrpE serum (1:10, 1:20) at 37 °C for 1 h, after which the EBL cells were infected for 1.5 h. Preimmunized serum served as a control. **E** The adhesion ability of *M. bovis* wild-type, *M. bovis* Δ*grpE*, and *M. bovis* Δ*grpE*:*grpE* to EBL cells was observed by laser scanning confocal microscopy. *M. bovis* wild-type, *M. bovis* Δ*grpE*, and *M. bovis* Δ*grpE*:*grpE* were infected into EBL cells, followed by staining with CFDA-SE, with the nuclei labelled using DAPI and the cell membrane marked with DiI. Red represents the cell membrane, green denotes *M. bovis*, and blue represents the nucleus.

The complementary strain *M. bovis* ∆*grpE*:*grpE* was successfully developed using the pIRR45-*grpE* complementary plasmid and identified by PCR (Additional file 3). Western blot analysis confirmed the expression of GrpE in the complementary strain *M. bovis* ∆*grpE*:*grpE* and the wild-type strain, whereas it was absent in the *M. bovis* ∆*grpE* strain (Figure 5A). Compared with those of the wild-type strain *M. bovis* PG45, the growth curves of these strains revealed that the lack of the *grpE* gene had a minimal effect on the growth of *M. bovis* (Figure 5B). However, the adhesion of *M. bovis* ∆*grpE* to EBL cells significantly decreased, whereas the adhesion of the complemented strain *M. bovis* ∆*grpE*:*grpE* exhibited a notable restoration of adhesion (Figure 5C). This finding suggests that the disruption of *grpE* gene adversely affects the adhesion of *M. bovis* to EBL cells. Additionally, these observations were corroborated by laser confocal microscopy analysis (Figure 5E). Before EBL cells were infected with *M. bovis*, anti-GrpE serum was incubated with *M. bovis*. The results demonstrated that compared with pretreatment with preimmune serum, treatment with anti-GrpE serum led to a significant reduction in the adhesion of *M. bovis* to EBL cells (Figure 5D). Importantly, the inhibitory effect of anti-GrpE serum was concentration dependent, indicating that higher concentrations of serum corresponded to greater reductions in bacterial adhesion. Taken together, these findings suggest that GrpE plays a vital role in the adhesion of *M. bovis* to EBL cells.

## Discussion

Adhesion to host cells is the first step in many mycoplasma infections. The binding of mycoplasma to host cells is essential for the initiation of infection and subsequent colonization [14]. Owing to their obligate parasitic lifestyle, the surface lipoproteins and various membrane-related proteins of mycoplasma play critical roles in adhering to host tissues, which are intricately linked to the pathogenesis of these organisms and are often regarded as the main candidate antigens for vaccine development [11, 29]. GrpE is highly conserved, which implies that it may be critical for the biological functions of mycoplasmas. In this study, we examined the role of GrpE in adhesion. GrpE is primarily associated with the membrane of *M. bovis,* and this surface localization suggests that it may be involved in crucial interactions with host cell membranes, potentially facilitating bacterial adhesion and colonization. By confocal microscopy analysis, our study revealed the adhesion of rGrpE to EBL cells and a significant reduction in adhesion following pretreatment with anti-GrpE serum, suggesting that GrpE plays a pivotal role in mediating the adhesion of *M. bovis* to host cells. These findings were further supported by the ELISA analysis, confirming that rGrpE binds to EBL cell membranes in a dose-dependent manner. Moreover, the function of GrpE in cytoadhesion at the bacterial level was investigated, and the results revealed that disruption of the *grpE* gene markedly impaired the adhesion of *M. bovis* to EBL cells, whereas the complemented strain *M. bovis* ∆*grpE*:*grpE* restored this ability, confirming that GrpE is indispensable for bacterial adhesion. Furthermore, treatment with anti-GrpE serum significantly inhibited the adhesion process of *M. bovis* in a concentration-dependent manner. Collectively, these results indicate that GrpE is a novel adhesion protein in *M. bovis.*

The host ECM is crucial for cell adhesion, and pathogenic microorganisms exploit interactions to facilitate their colonization on host cell surfaces, leading to infection [28, 30]. The interaction of rGrpE with ECM components, such as fibronectin, collagen IV, laminin, and vitronectin, was dose dependent, which indicated the multifaceted role of rGrpE in facilitating adherence to host cells and enhancing the survival and persistence of *M. bovis* within the host, which aligns with previous data that support the idea that bacteria often exploit ECM components to establish infections [11, 28, 31].

Plasminogen is a 92 kDa single-chain glycoprotein that undergoes proteolytic cleavage to form double-chained plasmin during the process of fibrinolysis in various animal species [32]. Certain bacteria express specific antigens, which interact with Plg, recruit Plg to obtain proteolytic activity, promote the invasion of pathogenic bacteria, or facilitate their distribution in infected animals [33]. Moreover, some mycoplasma species, including *M. bovis* [9, 14], *M. fermentans* [34], *M. gallisepticum* [35], *M. synoviae* [36, 37], *M. hyopneumoniae* [38]*,* and *M. pneumoniae* [39], have been shown to bind Plg on their cell surface and activate it to plasmin in the presence of tPA or urokinase plasminogen activator (uPA). Plg interacts with lysine residues on ligands through its kringle domain lysine binding sites. It potentially promotes bacterial adhesion and invasion by connecting Plg-binding proteins on the bacterial surface and host cells [40]. GrpE not only converted plasminogen to plasmin but also played a significant role in enhancing its activation to plasminase via the action of tPA, thereby influencing host fibrinolytic pathways. The inhibition of this activity by ε-ACA suggests that GrpE actively participates in modulating fibrinolytic activity in the host system, which may afford *M. bovis* a survival advantage within the infected host. This functional attribute further legitimizes the hypothesis that GrpE is an active participant in the pathogenesis of *M. bovis.* The effects of GrpE on plasminogen activation and binding to ECM components at the bacterial level will be studied in future research.

GrpE elicited significant antibody responses in *M. bovis*-infected and immunized calves but was not reactive to serum from healthy calves. The good immunogenicity of GrpE highlights its potential as a protective antigen, supporting the idea that GrpE may play a critical role in the immune response to *M. bovis*. It is highly conserved and expressed in wild-type strains and other clinical isolates, further reinforcing its potential as a candidate for vaccine development. Recent studies have reported that GrpE is a good vaccine candidate and provides protection against *Mycoplasma synoviae* infection [41]. These findings further underscore the potential of this surface-exposed protein as a novel immunogen for vaccines and therapeutic intervention.

In summary, the chaperone GrpE serves as a multifunctional protein capable of adhering to host cells in *M. bovis*. GrpE interacts with the host ECM and plasminogen, contributing significantly to the pathogenesis of *M. bovis*. GrpE is highly conserved and may be a promising vaccine and drug target for the prevention and control of *M. bovis* infections.

## Supplementary Information

**Additional file 1:** ***M. bovis*** **strains used in this study.** List of *M. bovis* strains used in this study.

**Additional file 2: Expression, purification, and identification of rGrpE.** **A. **SDS-PAGE analysis of rGrpE purified on a Ni‒NTA column. **B **Western blot analysis of GrpE was performed with anti-GrpE serum (1:2000) and anti-6×His tag monoclonal antibody (1:10 000).

**Additional file 3:** **PCR identification of the grpE gene in the** ***M. bovis*** **∆grpE strain and its complementation strain.** PCR-based identification of the *grpE* gene in the *M. bovis* wild-type strain, *M. bovis *∆*grpE* strain, and complementation strain *M. bovis *∆*grpE*:*grpE*.

## Data Availability

The datasets analysed are available from the corresponding author upon reasonable request.
