# Supplementary material for: The chaperone GrpE mediates adhesion in Mycoplasma bovis and interactions with host extracellular matrix components and plasminogen
Source: Vet Res. 2025 Oct 29;56:205. doi: 10.1186/s13567-025-01619-4 (PMC12574120; doi:10.1186/s13567-025-01619-4)
Supplement: Supplementary file 1 — Additional file 1: M. bovis strains used in this study. List of M. bovis strains used in this study. [file 13567_2025_1619_MOESM1_ESM.docx]

**Additional file 1 *M. bovis* strains used in this study**

| **Strain** | **Host** | **Site** | **Country** | **Year** |
| --- | --- | --- | --- | --- |
| PG45 | cow | milk | USA | 1961 |
| 08M | cattle | lung | China | 2008 |
| 07801 | cattle | lung | China | 2010 |
| P-1 | yak | lung | China | 2020 |
| 1XS | cattle | pleural effusion | China | 2010 |
| 0709 | cattle | lung | China | 2010 |
| 0794 | cattle | lung | China | 2010 |
