# Supplementary material for: The chaperone GrpE mediates adhesion in Mycoplasma bovis and interactions with host extracellular matrix components and plasminogen
Source: Vet Res. 2025 Oct 29;56:205. doi: 10.1186/s13567-025-01619-4 (PMC12574120; doi:10.1186/s13567-025-01619-4)

A

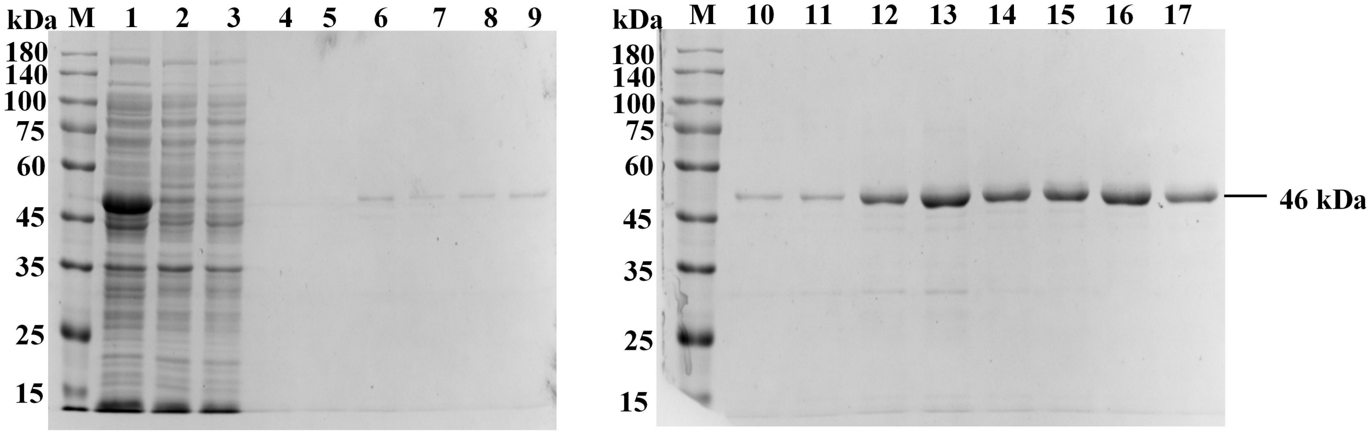

M:Marker;1:induced bacterial lysate;2:flow-through supernant;3-4:20 mM imidazole concentration; 5-6:60 mM imidazole concentration;7-8 :80 mM imidazole concentration;9-11:100 mM imidazole concentration;12-14:200 mM imidazole concentration;15-17:300 mM imidazole concentration

B

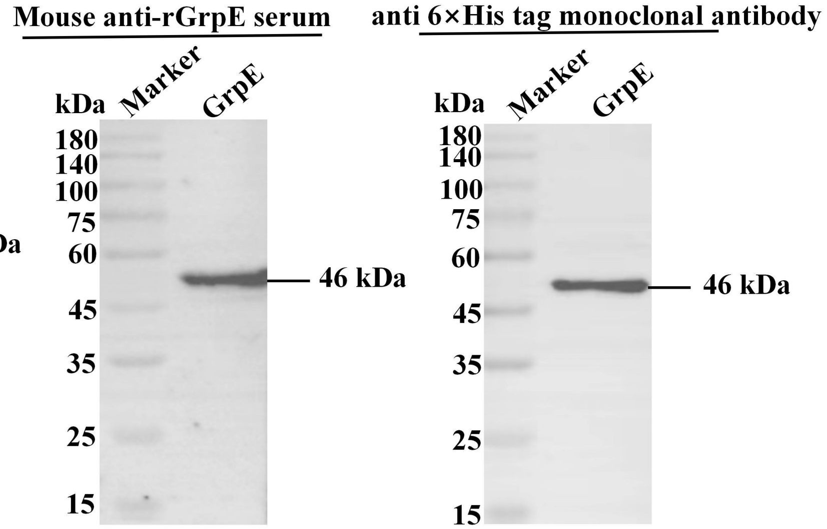

Supplement: Supplementary file 2 — Additional file 2: Expression, purification, and identification of rGrpE. A. SDS-PAGE analysis of rGrpE purified on a Ni‒NTA column. B Western blot analysis of GrpE was performed with anti-GrpE serum (1:2000) and anti-6×His tag monoclonal antibody (1:10 000). [file 13567_2025_1619_MOESM2_ESM.pdf]
