# Supplementary figures and images for: The chaperone GrpE mediates adhesion in Mycoplasma bovis and interactions with host extracellular matrix components and plasminogen
Source: Vet Res. 2025 Oct 29;56:205. doi: 10.1186/s13567-025-01619-4 (PMC12574120; doi:10.1186/s13567-025-01619-4)

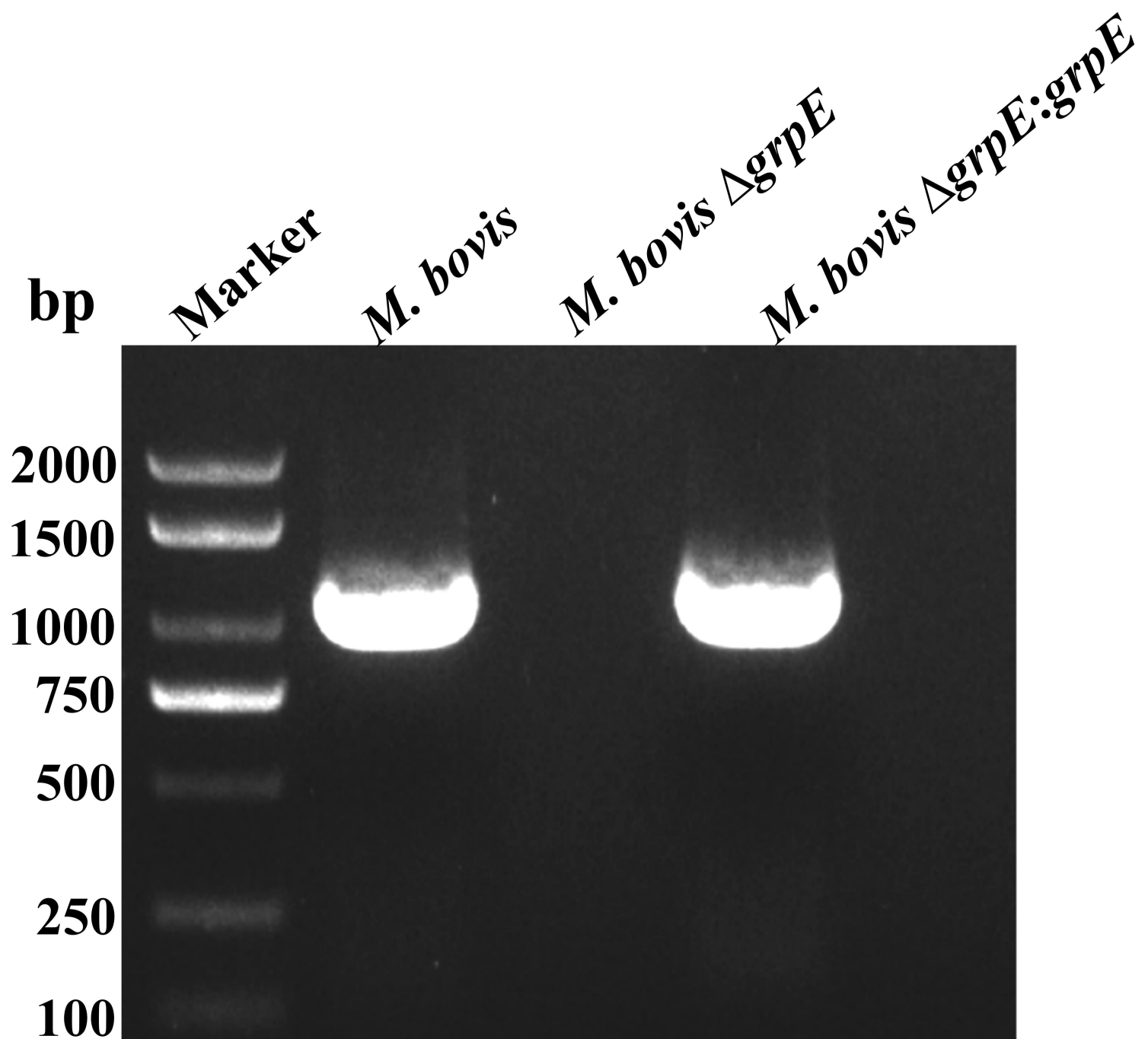

Supplement: Supplementary file 3 — Additional file 3: PCR identification of the grpE gene in the M. bovis ∆grpE strain and its complementation strain. PCR-based identification of the grpE gene in the M. bovis wild-type strain, M. bovis ∆grpE strain, and complementation strain M. bovis ∆grpE:grpE. [file 13567_2025_1619_MOESM3_ESM.pdf]
